# Feminist retroviruses to white Sharia: Gender “science fan fiction” on 4Chan

**DOI:** 10.1177/09636625241228160

**Published:** 2024-02-27

**Authors:** Nicole Iturriaga, Aaron Panofsky, Kushan Dasgupta

**Affiliations:** University of California, Irvine, USA; University of California, Los Angeles, USA; University of Louisville, USA

**Keywords:** public understanding of science, representations of science, rhetoric of science and technology, science and popular culture, science fan fiction, social movements

## Abstract

This article demonstrates—based on an interpretive discourse analysis of three types of memes (Rabid Feminists, Women’s Bodies, Policy Ideas) and secondary thread discourse on 4chan’s “Politically Incorrect” discussion board—two key findings: (1) the existence of a gendered hate based scientific discourse, “science fan fiction,” in online spaces and (2) how gender “science fan fiction” is an outcome of the male supremacist cosmology, by producing and justifying resentment against white women as being both inherently untrustworthy (politically, sexually, intellectually) and dangerous. This perspective—which combines hatred and distrust of women with white nationalist anxieties about demographic shifts, racial integrity, and sexuality—then motivates misogynist policy ideas including total domination of women or their removal. 4chan users employ this discourse to “scientifically” substantiate claims of white male supremacy, the fundamental untrustworthiness of white women, and to argue white women’s inherent threat to white male supremacist goals.

We typically think of science and hate as being combined to assert racial hierarchy, naturalize domination, and so on (for more on this, see Panofsky et al., 2021). This article argues that 4chan users also employ *gender science* theorization in message boards to assert patriarchal hierarchies, domination, and articulate resentment. We demonstrate, based on an interpretive discourse analysis of three categories of memes and secondary thread discourse on 4chan’s “Politically Incorrect” discussion board, that 4chan users engage in what we call gender *science fan fiction* to “scientifically” theorize gender and express frustrations about white women’s unreliability. This means that users take scientific findings and ideas with no relationship to gender, like research on Alzheimer’s, and then link it to misogynistic and racist concerns not part of the original scientists’ intent. From there, they go on to combine, remix, and extrapolate baroque conclusions that are then used as evidence for the fight against white women’s inherent treachery and the need for a white ethno-state—thus, reinterpreting and inferring new outcomes loosely based in the universe of the original science.

In traditional fan fiction authors start with the materials of a pre-existing fictional world and then obey some of that world’s narrative rules while bending, breaking, and remixing others, creating new stories and narrative possibilities that push the original authors’ intentions in new directions (Hellekson and Busse, 2014). So too with “science fan fiction”: 4chan gender theorists take concepts, findings, and figures from various scientific publications, and then recombine and reinterpret them in ostentatiously fantastic ways to theorize regressive gender ideas—particularly about white women’s supposed inherent treachery and their betrayal of the race.

Intriguingly, part of what makes gender theorization through “science” fan fiction a distinctive type of science misappropriation is that it mostly avoids the pre-existing stock of misogynistic science. For example, it does not use scientific literature claiming essential gender differences in spatial reasoning, intelligence, evolutionary sex roles, or “deceitful” mating strategies, all of which are readily available (Saini, 2017). This sharply contrasts with far-right appropriations of non-gendered, racial research, in which much of the discourse involves discussion and amplification of the pre-existing corpus of professional scientific racism or decontextualization of claims and images from medical and population genetics research related to race (Carlson and Harris, 2020; Panofsky et al., 2021).

This article demonstrates the following two key findings: (1) the existence of a *gendered* hate-based scientific discourse, “science fan fiction,” in online spaces and (2) how gender “science fan fiction” is an outcome of the male supremacist cosmology, producing and justifying resentment against white women as being inherently untrustworthy (politically, sexually, intellectually) and dangerous. This perspective—which combines hatred and distrust of women with white nationalist anxieties about demographic shifts, racial integrity, and sexuality—then motivates misogynist policy ideas including total domination of women or their removal. 4chan users employ this discourse to “scientifically” substantiate claims of white male supremacy, the fundamental untrustworthiness of white women, and their inherent threat to white male supremacist goals.

## “Science” and fan fiction

Research has recently noted a rise in “scientific racist” discourse among the far-right on the Internet (Carlson and Harris, 2020). This literature demonstrates that white nationalists purposively take and misuse the findings of anthropologists, human geneticists, and other scientists, as well as sometimes produce their own pseudo-research. All of this is done to demonstrate that race is genetic rather than social, genes produce racial differences in behavior, culture, and cognition, and thus that some racial groups are genetically bound to have preferable social outcomes (Panofsky et al., 2021).

Unnoticed within this phenomenon is what we call “gender science fan fiction.” Gender science fan fiction is connected to this racial science through entangled themes. Users draw on science to ground ideas about racial separation and white supremacy within notions of male domination. They do so by constructing men as crucial to the leadership of whites’ racial interests and creating a scientific case for strict patriarchy.

Where gender science fan fiction departs is in structure and practice. None of the content we analyze in this article involves the selection and amplification of sex differences research from the peer-reviewed academic literature, to argue that women are biologically unfit for leadership and advancement of whites’ racial interests. Instead, users develop utterly peculiar scientific accounts, drawn from research unrelated to sex differences. Concepts from this unrelated research are re-theorized in often orthogonal ways to construct white women as untrustworthy, treacherously undermining whites’ racial interests, and therefore in need of domination.

In doing so, users exhibit a parallel style of theorization commonly found in fan fiction literature. Such stories “often take the pre-existing story-world in a new, sometimes bizarre, direction” that reflects the authors’ ideological interests (Thomas, 2011: 1). Similarly, gender science fan fiction uses pre-existing scientific concepts to go in bizarre directions that re-theorize biology’s relationship to gender or nature’s case for patriarchy.

Traditionally, fan fiction refers to stories created by fans centered on characters and plots from pre-existing canons of work or single source texts. For this reason, we need to clarify how our concept of “science fan fiction” departs from some prior understandings and analyses. First, fan fiction is as much about the act of fictioning as it is about the fandoms or communities that accompany the activity. Given our focus on elaborating an unnoticed form of science appropriation, we concentrate more on the meanings and actions associated with this creative activity rather than substantive descriptions of the white nationalist or far-right sympathizers who populate message boards.

Second, many understand fan fiction as involving the production of lengthy narrative forms, from short stories to entire novels. Yet, as others note, the creative repurposing encapsulated by fan fiction can involve more synoptic forms, such as single message board posts to images. We thus focus on memes. Internet memes are semantic information that combine pictures, hashtags, text, or video, often adapted, or redesigned in a way to fit an identity (Guenther et al., 2020). Internet consumers diffuse memes through personal networks by commenting, sharing, or liking them (Spitzberg, 2014). Memes are especially useful for political communication and expression between and within right-wing groups (Klein and Muis, 2019), as they function as mental and visual depictions of political ideologies, establishing a shared social and moral order that shapes other content creators’ worldviews (DeCook, 2018: 489). The far-right is particularly adept at creating armies of memes that embed ideological messages in online spaces (Colley and Moore, 2022).

Third, though much of the literature understands fan fiction to be democratic, feminist, and positive, our findings parallel research on “toxic fandoms.” This research indicates the same techniques of transmedia storytelling, participatory culture, community, and belonging can anchor fan communities in toxic ideologies (De Zeeuw and Gekker, 2023; Stanfill, 2021). Thus, fan communities, such as those discussed here, which center on misogyny, racism, white supremacy, and other troubling ideologies have long existed (Hodson and Gosse, 2022; Reinhard et al., 2022; Stanfill, 2020). Other research has analyzed the far-right conspiracy theory QAnon as a fandom and safe space for the expression of inappropriate beliefs (Hodson and Gosse, 2022; McMillin, 2020); and find that its use of fandom techniques create direct bridges to “other extremist ideological communities, like white nationalists, due to overlapping feelings, beliefs, and ultimately identities” (Reinhard et al., 2022: 1165). We contribute to this growing field, by demonstrating toxic fandoms on 4chan, centered on white misogynist supremacy, employ “scientific texts” as canon for participatory storytelling centered on white women’s treachery. We further illustrate how this is done through shared memetic imagery and discussion posts.

## 4chan and the far-right online

4chan began in 2003 as a discussion board website that allows users to post anonymously. It has over 40 distinct message boards on different topics where content is prolific and temporary (Kasimov, 2021: 152). 4chan remained on the fringes of the Internet until the 2014 GamerGate controversy, which, in sum, began when female video game aficionados brought feminist critiques to the gaming community. The women involved including the original feminist gamers, female journalists who covered the story, and other gamers who spoke about the controversy—were doxed and threatened with rape and murder, much of which was facilitated by 4chan (Mortensen, 2018). Scholars situate the controversy as *the* moment when 4chan’s political tendencies solidified as the place for far-right politics (Atkinson, 2018).

GamerGate is also when the “politically incorrect” board became the site’s most popular and an active recruiting zone for white supremacist organizations (Phillips, 2018: 23). Today, the /pol/board accounts “for approximately 20% of all traffic to the site” and attracts around 22 million users a month (Kasimov, 2021: 152). Research on the /pol/board has shown that various far-right communities including Incels (involuntary celibates), men’s rights groups, and white nationalists frequent the space, and is full of far-right recruitment tactics “via pseudo-science, propaganda . . . and decontextualized statistics” (Bessant et al., 2021; Kasimov, 2021: 157; Zelenkauskaite et al., 2020).

Although there has been research on 4chan (Hine et al., 2017; Kasimov, 2021), there is little on scientific memes. This article adds to this discussion by demonstrating how these groups theorize and contextualize their values about racial and patriarchal hierarchies through scientific misogyny. Explicitly, it highlights how misogynistic science theorization dramatizes resentment of white women and the desire to control the reproduction of both the individual and political body. It also illuminates how this discourse creates common ground among different factions in 4chan cultures who share grievances about women, whom they frame as being inherently treacherous creatures across all categories (political, biological, sexual).

Research on the far-right online more generally has taken different perspectives such as arguing that these websites function as a “safe space” for like-minded groups and fandoms (Caren et al., 2012; Reinhard et al., 2022). Others have argued that they function more as a loci for “communities” to “discuss strategic considerations, plan events on—and offline, build collective identity, incite individual or collective action and recruit new members into the movement” (Kasimov, 2021: 15). Nonetheless, many agree that the creators of these message boards target young men engrossed in Internet culture with the intent to radicalize (Marwick and Lewis, 2017). Men attracted to these virulent spaces often feel betrayed by traditional power structures. They blame their perceived loss in status and power on “undeserving” minorities and express the hope of returning to a world where white men are in control (Gotell and Dutton, 2016: 487). Research on “the manosphere” has demonstrated that though far-right men may possess different agendas, they unite around “the common goal to defeat feminism or keep women out of the space” (Ging, 2019: 653). One key aspect of this type of radicalization is redpilling, or the delivery of a discrete set of facts and ideology that will produce a gestalt switch to awaken men from the mental jail of liberalism and feminism (Stern, 2019).

However, the extremist-right’s use of the Internet is not new, as it has long been a diffuser for both right-wing ideological materials and general hate speech (Daniels, 2018) and an important white nationalist terrorist organizing venue (Holt and Bolden, 2014). Yet, with the rise of anonymous web forums like Reddit, 4chan, and 8kun, more virulent forms of extremist ideology are proliferating across the Internet.

## 1. Site and data collection

This project began with an interest in how the far-right produces, posts, and discusses memes related to gender science. Thus, 4chan’s “politically incorrect” board was the obvious venue. Most threads start with a user posting an image or a meme and a title. Other users then respond to the originating image and each other. All 4chan posts are anonymous and content is automatically erased, sometimes within hours. We accessed content by using the site 4plebs.org, an unofficial 4chan archive site. Furthermore, rather than scan the totality of 4chan to discern most frequent content, we prioritized building a well-selected corpus of content (Recuber, 2017). We based this on our initial research interest in far-right *practices* involved in the production and discussion of gender science memes, and the need for a targeted set of materials that could be subjected to detailed qualitative analysis. We began by collecting threads produced by search terms related to “women,” “feminism,” and “reproduction” within the years of 2018–2020 and analyzing for memes. The final memes chosen were among the most used gender science memes (a frequency of at least six times a year), clearly exemplified the discourse of scientific misogyny, and created large discussions. For all three selected memes, we collected *N* = 45 threads ranging from 3 to 55 pages. Our approach led to the discovery of science fan fiction, linking to other literature on far-right discourse (Colley and Moore, 2022; DeCook, 2018), but it does not allow us to quantify its prevalence.

We conducted our analysis and coding by drawing on the abductive analysis literature (Timmermans and Tavory, 2012). We expected the gender science memes to reflect traditional scientific misogyny and efforts to popularize such ideas. While such content did emerge, we quickly noticed scientific discourse that did not reflect previous understandings of scientific misogyny. These memes were baroque and seemingly otherworldly in their conclusions about gender all while using scientific concepts and imagery. We sorted the memes into a separate category, to better understand the differences in relation to traditional scientific misogyny and subject them to further categorization coding.

We analyzed this content with an interest in the memes’ knowledge and cultural practices, the memes’ imagery and textual discourse looking specifically at the presented arguments, use of academic citations, and images. Correspondingly, we analyzed thread discourses for key ideas such as science, white nationalism, scientific discourse, and hate speech. We also coded for “shit posting” a form of harassing or trolling that are deliberately meant to amuse and shock, perceived attempts at humor or sarcasm, and other nuances of 4chan culture. Early on, two dimensions stood out. First, the memes incorporated a particular style of citation and appropriation practice, as the citations led to literature with no relation to gender but were used to substantiate misogynistic ideas. Second, rather than—or, in some cases, in addition to—emphasizing women’s inferiority, these memes aimed to make some point about women’s untrustworthiness. By reflecting on these themes, our theorization of this content as a kind of science fan fiction began.

In what follows, we focus on memes that demonstrate science fan fiction. Although these categories may not characterize the entirety of the genre on 4chan, they are illustrative for this article’s purposes, as they make salient key dimensions of gender science fan fiction. All quotes were transcribed directly with errors intact.

## 2. Findings

Below, we analyze memes organized around three themes—Rabid Feminists, Women’s Bodies and Reproduction, and Policy Ideas. Throughout the analysis, we first unpack the primary discourse of the meme and then the secondary discourse of thread posts. Our analysis shows that users utilize science fan fiction to express resentment and advance theoretical conversations including dilemmas about purity, the white ethno-state, and white women’s role in reproducing the white nation. Through these discourses, 4chan functions as a political forum where users conceptualize their political obstacles (i.e. feminism), reaffirm white male supremacy, and fetishize sexual ideal types (i.e. virgins). 4chan in general is a speculative space where larger conversations range from debating Nazi theories to pragmatic approaches to creating a white ethno-state.

Importantly, we followed each of the academic citations in the memes. Almost all were real scientific articles. *None* of the papers cited corresponded to the scientific conclusions attributed to them. The memes, thus, did not function to promulgate pre-existing scientific ideas, rather they were a jumping off point for novel, often baroque, misogynist theorizations, and assertions—the process we call science fan fiction.

## 3. Rabid feminists

**Figure 1.**
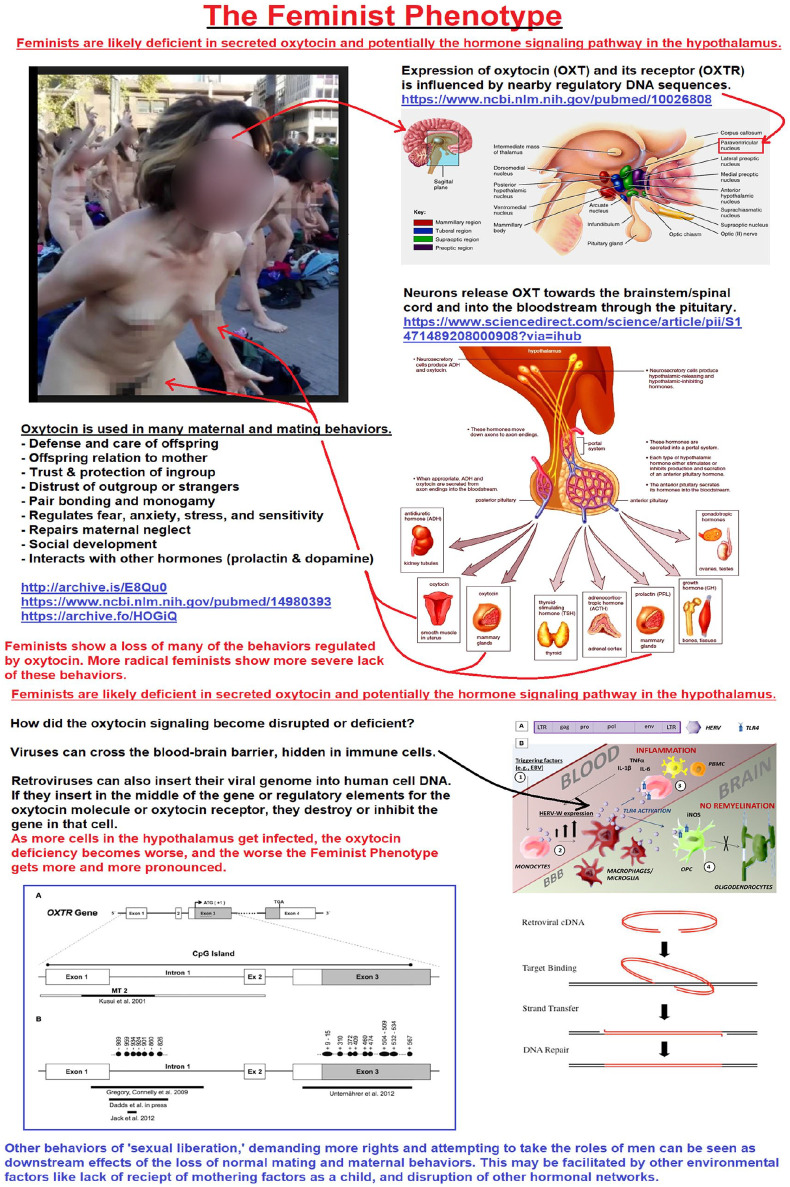
Feminist Retrovirus Meme.

Figure 1 focuses on the hatred of feminism, a common locus for shared belief, and purports that feminism is caused by a retrovirus.

It claims that feminism is a retrovirus that attacks the brains of women, so they are unable to produce or receive oxytocin—the hormone often associated with empathy or maternal bonding. The virus causes the belief in feminism and produces the feminist phenotype making infected women physically unattractive. It also forces women to engage in sexual liberation, demand civil rights, and take the roles of men, which, “can be seen as downstream effects of normal and maternal behaviors.” The imagery of this meme is extremely science-y with graphs, charts, and jargon that explains the virus.

Figure 1 illustrates a particular approach to what 4chan users call the “woman problem”—a shorthand for why white women fail to comply with traditional sex roles, assert their own rights, and resist men’s sexual advances. The citations were real studies that focused only on the impact of oxytocin on maternal bonding. The claims advanced in the meme are not drawn from the scientific literature, but represent a pseudoscientific fantasy pieced together from scientific loose ends.

### Science talk

Figure 1 encouraged “science talk” among thread comments where users contributed their own scientific ideas about the virus. In one, a user responding to the meme posted,

A bioweapon, to be exact. Destroying every society where Femin is unleased. Women with lower oxytocin and the inability to pairbond will continue to hate marriage and motherhood, slowly deteriorating Family until society is so immoral & dysfunctional that it is overrun.

Here, the user expanded the original text to propose the virus is a bioweapon that tricks women into hating marriage and motherhood, ultimately creating immoral and dysfunctional societies. This explanation places no responsibility on men, who are seemingly immune to the virus, for the degradation of society.

In other cases, commentators’ expansion of the meme’s science took a decidedly white nationalist tone. In a different thread, entitled “Feminism Bioweapon,” one user posted,

How to spot infection!

Wanting to destroy the family and men, hatred of children, wanting (and doing) killing of babies along with abortion, sexual/gender confusion, increase rates of homosexuality, pedophilia, bestiality, necrophilia, transgender, various disturbing fetishes, cannibalism, wanting to destroy whites, support open borders . . . Any woman that is feminist is FV infected. Any woman that is anti-(white) male is FV infected . . .

Here, the commenter incorporates white nationalist discourse by including new symptoms, which combine progressive tenets such as support of the LGBTQIA+ community, along with necrophilia, cannibalism, and “wanting to destroy whites.” It is not possible to know if these commenters believe their claims, are making jokes, or are being as outrageous as possible for fun. Regardless of intent, these comments demonstrate how users can take an already baroque biologization of feminism and further expand it into wild claims and incorporate white nationalist themes, like the idea that feminists are diseased and therefore inherently untrustworthy. It also demonstrates the use of “science” to articulate resentment against white women for their treachery.

### Political discussions

Others used Figure 1 to launch extended political imaginings of their larger movement. In some instances, the science of the meme was connected to solving the “woman problem” through removal. For example, one post said, “Personally, I think we have to move beyond women. We can use the artificial womb to have children and prostitutes/sex robots can. Be used for sex.” Others continued, saying, “white women need to be replaced by science. Science has found many solutions to the worlds problems (e.g. disease). White women are the disease and science will find a cure.” Another commented, “I’m leading towards genocide.” In this case, the science fan fiction of the memes segued into what users saw as the bigger issue at hand, the existence of all women (though perceivably more focused on white women), their inherent untrustworthiness, and its correlation to the destruction of the white race. From here, the discourse moved into how science could “cure” this problem with artificial wombs, replacing the biological need for women. As will be seen, the idea of woman-free reproduction or enslaving of women comes up as a solution to several misogynistic fantasies.

For others, Figure 1 provoked calls for racist eugenics policies and redpilling data dumps—or links to more “scientific” articles that led off platform. For instance, a user posted, “We need to support positive and negative eugenics so the intelligent and honorable are bred not big dick n******.”^1^ Later, in the same thread, another user argued that the thread needed the “next level of pills” and data dumped 16 individual links to “scientific” articles about “femin” the bioweapon. The conversation continued, debating whether white women are inherently evil, are working alongside Jews, and idealization of virgins.

At other times, it was used to connect white women and feminism with racist and antisemitic ideas. In one thread, the feminist virus meme was deployed alongside the question, “can we even save white women.” Another user responded, “Women are the jews of gender,” quickly followed by, “The Woman Cries out in Pain as She Stabs you in the Back. The Jew Cries out in pain as He Stabs you in the Back.” The imagery of being stabbed in the back is a classic antisemitic trope. Its rearticulation to include white women is significant to understanding 4chan users’ development and use of gender science fan fiction, as they are essentially arguing that the true enemy to the white race is white women. White women are the enemy within due to their innate disloyalty, whether it be political (feminism), sexual (withholding sex from whites/having sex with non-whites), or biological (will be discussed later).

Importantly, these examples demonstrate how 4chan users’ engagement with gender resentment builds new scientific realms, unconnected to academic science, to explain why white women are fundamentally devious, and how they, as endangered white men, need to respond. The different applications (removal to antisemitic tropes) may also reflect how the various far-right communities that make up 4chan—white nationalists to Incels—can find overlap in science fan fiction rhetoric as a locus and means of shared grievance.

The scientific concepts of gender science fan fiction may seem fundamentally different from the 4chan users’ treatment of race science, which amplify pre-existing professional scientific racism or decontextualized claims from medical and population genetics to advance a “race realism” argument. Yet, upon closer examination, both discourses are intrinsically connected in that they exemplify the movement’s beliefs about white supremacy, the need for racial separation, and strictly enforced patriarchy. Consequently, the two approaches both signify a difference in the use of the scientific literature and fictional imaginations in the service of rearticulating a racialized gender politics.

## 4. Women’s bodies and reproduction

A large portion of gender science memes on 4chan are straightforward misogyny, which become complicated by science fan fiction concepts. This genre of memes scientifically explains why women are biologically inferior to men and open to contamination.

### Biological inferiority

**Figure 2.**
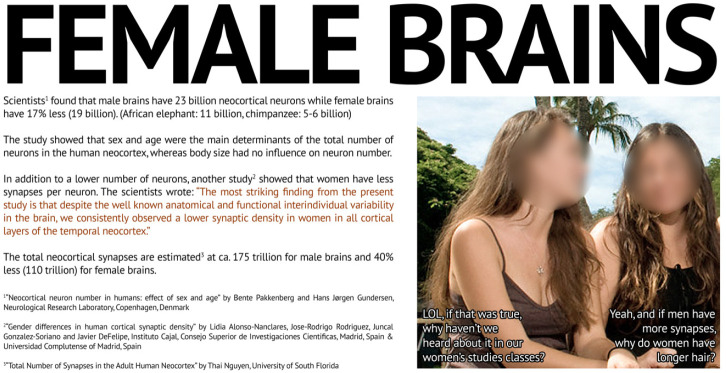
Female Brains Meme.

Figure 2 fits into long-standing misogynistic ideas about the physical differences between men and women. Specifically, the meme’s “science” suggests that women have inferior brains due to the lack of density in their synapses. The meme hits typical 4chan contempt for higher education, women’s studies, and women, with the inclusion of a photograph of two long-haired, smiling white women. The caption underneath alludes to their suggested conversation as one says, “LOL, if that was true, why haven’t we heard about it in our women’s studies class?” The other responds, “Yeah, and if men have more synapses, why do women have longer hair?”

All provided citations were from scientific studies focused on demonstrating that Alzheimer’s and other forms of dementia decrease the number of synaptic brain connections. None focused on gender differences.

### Science talk

Although Figure 2 is not explicitly racialized, its appearance in a thread almost always elicited racist responses or alluded to ideas about keeping racial lines pure. For example, one commenter engaged in science talk while hitting far-right talking points:

ah, that’s actually the next step in this progression of pills, when you look at reproduction like this . . . when a woman has a daughter, there are 3 X [chromosomes] competing yet room for 2. Mom has an X from grandma, and one from dad’s mother . . . which one did the daughter get? Women only have 50% chance of reproducing the matrilineal organism when they have daughters, this means that the matrilineal organism will evolve slower than the patrilineal one, which is why women have 40% less synaptic density than men yet have the same number of braincells while seeming completely useless in every conceivable way across all time.

you track boys, you track virgins, that’s how it’s done because that’s how it works. Everything else is lossy and mixed. This is also why marriages must be arranged, to protect lineages and ethnicity, family lines.

The commenter expands the “science” of the meme to further argue the biological inferiority of women by imagining a selfish gene logic of evolutionary competition among chromosomes, then making a logical leap to women having less synaptic density. This lack implies that women are less intelligent than men. They also connect the meme’s assertion that human women only have a few more brain synapses than African elephants and chimpanzees due to matrilineal lines’ slow evolution. This meme provides another baroque example, as they revel in complicated thinking about biological sex differences but are disconnected from the science it is claiming to explain.

Importantly, these perceived biological differences lead to the argument that women are “completely useless in every conceivable way,” indicating the need for arranged marriages with virgins to protect white blood lines or else people become “lossy and mixed.” The fundamental assumption being white women, if left to their own devices, will betray the white race. Moreover, this betrayal may not be intentional, but rather a consequence of dimwitted ladies whose biology forces bad decisions without conscious understanding. This is one variation of white women’s untrustworthiness. This perspective also provides a reason for the fixation on virgins, as the ideal type, and how this preoccupation makes its way into scientific explanations of both male superiority and white supremacist imaginings of blood line purity. Notably, the meme does not come off as overtly racist or far-right, yet it spurred comments like the quote above that heavily leans into white nationalist imaginings.

### Trojan horses

**Figure 3.**
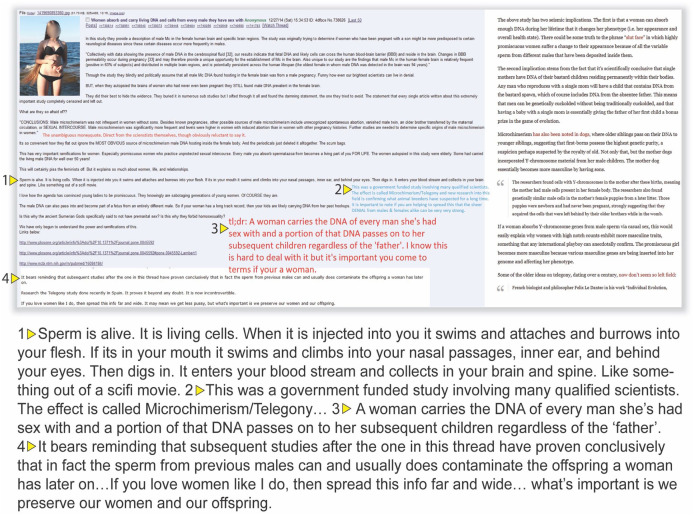
Microchimerism Meme.

The key scientific concept of Figure 3 is microchimerism, a real realm of scientific inquiry that is misconstrued here. In actual microchimerism studies, scientists investigate the incorporation of DNA from a fetus into the mother’s body. The meme, however, inaccurately describes microchimerism as occurring when women’s DNA becomes permanently affected by the sperm of male sexual partners. The claim is that this causes women’s future children to carry a mix of the DNA of past sexual partners in addition to that of the fetus’s biological father.

The science fan fiction of Figure 3 is thus a mixture of two scientific concepts that are misapplied. The first is the idea of a mother’s incorporation of a fetus’s DNA. The second is that a woman can have children with mixed paternity. This seems to derive from superfecundation, which is found in other animal species like dogs. For example, when a female dog is in estrus, she releases many eggs. During this 2- to 3-week cycle, it is possible for the female’s litter to have multiple fathers. The meme creates a hybrid concept of microchismerism and superfecundation related to unprotected sex.

Figure 3 also reifies male power through the strength of sperm, as it says, “Sperm is alive . . . It enters your blood stream and collects in your brain and spine.” The focus on the supposed neurological impacts of microchimerism offers a new explanation for the supposed behavioral inscrutability of women, another variation of untrustworthiness.

Figure 3 also connects the “science” to political arguments against feminism, premarital sex, and “loose” women who are “sabotaged.” It ends by suggesting further investigation into telegony, a hereditarian theory that argues progeny can inherit features from previous sexual partners of the female parent, claiming that science has proven it exists “beyond any doubt. It is not incontrovertible.” Of course, the underlying assumption is that males, as superior beings, do not face the same dangers of receiving or being changed by their female partner’s DNA. Nor is there any suggestion that male-to-male sexual contact could potentially change a partner’s future sperm.

All the citations are from scientific papers that solely focus on finding male DNA, of presumed fetal origin, in female bodies years after delivery and its potential to create autoimmune responses during and after pregnancy. None of the citations suggests or discusses the idea that the DNA of previous lovers’ impact future children’s DNA.

### Science talk

As with the previous memes, some of the secondary discourse focused on expanding the science. Intriguingly, several of these conversations specifically connected microchimerism with Figure 3, illustrating a curious overlap of science fan fiction theorizations. For example, one post said,

odd we have been sharing such testing for over a year that shows that Its [microchimerism] true . . . virgins are the only way to make a true heir, and this scientific fact really hurts your feelings . . . the oxytocin imbalance leads women to destroy their host nation, their in-group’s dominance, destroy their children.

This comment reflects an idealization of virgins, who are touted as the only path to pure children, both racially and genetically. It further suggests that white women are much like Trojan horses—opposed to hyper-patriarchal beliefs, diseased (presumably with Femin), and ready to destroy their “host nation” and their children.

As seen before, the secondary discourse ranged from discussions about the inherent untrustworthiness of white women to their perceived desire to race mix. The meme also elicited conversations about why the white ethno-state is needed, how to tell if a child is carrying non-white genes, why one should only marry virgins, and the need to be wary of white women who have had sex with non-white men, as their future children will not be white. One commenter argued that microchimerism was part of the Jewish conspiracy saying,

Streicher (the DerSturmer director) said this in 1933 . . . alien albumen is the sperm of a man of alien race. The male sperm in cohabitation is partially or completely absorbed by the female, and thus enters her bloodstream. One single cohabitation of a Jew with an Aryan woman is sufficient to poison her blood forever . . . Never again will she be able to bear purely Aryan children . . . They will all be bastards, with a dual soul and a body of a mixed breed . . .

This post illustrates how a thread’s discourse can include more theoretical arguments based in Nazi theories about Jewish contagion and the ease of toxifying women. These comments also highlight the fetishization of virgins, as they are the only uncontaminated option.

At other times, users would reject theoretical arguments in favor of pragmatic action. For example, one user posted,

Out here in the PNW [Pacific Northwest] of the US we have separatist movement that’s starting up again. We’re taking any and all white men or women of good character, just as the founding fathers intended. Many of the people who find us are damaged and broken . . . We want women who will look for white men within this movement, who can have white children and raise them well. If they can’t have children, they will have to offer something else . . . A hymen will not save the white race.

Here, we see a clear division between those fleshing out white purity theories versus those who are moving into action, such as building a white nationalist community. This user does not care about the reproductive purity of white women; what matters is women’s commitment to serve in the patriarchal white nationalist order. Thus, for some, the conversation is about theoretical debates about purity and contagion. For others, it is about pragmatic action.

The theme of removal ran across multiple discussions. One thread involving Figure 3 focused on whether science could remove female reproduction and, if so, whether to eliminate women altogether or turn them into sex slaves. This included a discussion about using transwomen as superior sexual partners:

[When] trannies get fully working, perfected vaginas, no one will choose a real woman. Why would you want a female with an inferior manipulative female mind when you could have female (male) with a male brain . . . artificial womb tanks plus perfect male to female trannies equals humanity getting rid of the inferior, obsolete, original female.

In this and other examples, we see how following the theoretical concerns with purity and the imagination of technical solutions to a logical conclusion leads some to pursue ideas (i.e. sex with transwomen) that in ordinary contexts would be utterly taboo. These types of suggestions, though not the norm, were found regularly in thread discourses. However, other users would quickly end these discussions with homophobic, racist, and misogynistic taunting.

As such, across examples, we see different approaches and responses to the idea that white women are biologically untrustworthy. In some threads, there is a push toward denigrating white women as being impure and attracted to other races. In others, there is a tension between the theorists, like those who reference Nazi science, and those who value the pragmatics of action, such as wanting to begin the white ethno-state in the Pacific Northwest. While in others, there is a return to the suggestion of removal, but with the added alternative of using transwomen as sex slaves. Yet, across these examples, what remains constant is the use of gender science fan fiction to bolster claims that prove the biological treachery of white women while searching for solutions to the “problem.” The pragmatic path says this is a pseudo problem, the theoretical courts political taboos. Either way, these discussions radicalize the threads’ discourses, allowing far-right talking points to come into focus.

## 5. Policy

This last grouping looks at policy science fan fiction memes and how they both fetishize virgins and uphold extreme patriarchal ideas.

**Figure 4.**
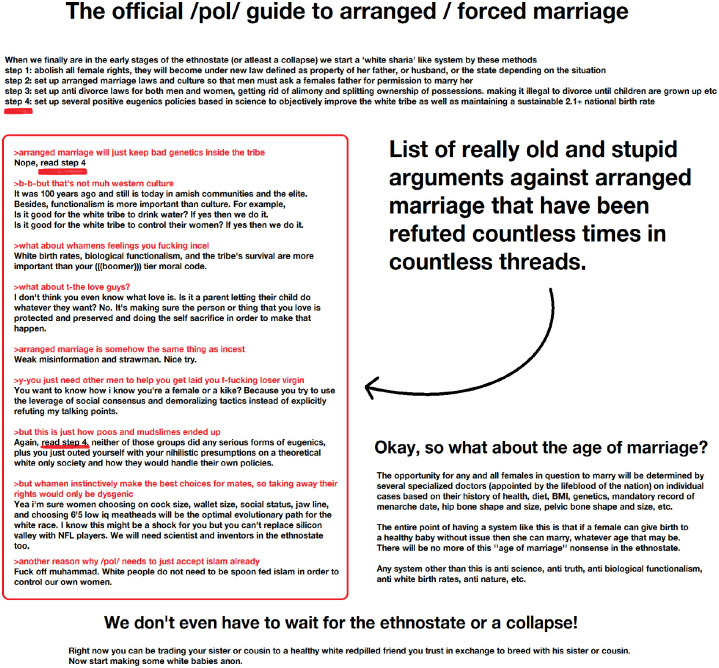
Policy Meme.

Figure 4, unlike the others analyzed, focuses on policies meant to prevent white genocide without necessarily having an ethno-state in place. The author bases recommendations on perceptions of Sharia law with white nationalists as the controlling power. Although it does not have scientific citations, the meme refers to past and future “scientific” efforts as the basis for ideas. For example, in the first line, step 4 says, “Set up positive eugenics policies based in science to objectively improve the white tribe.” In addition, they argue that “Any system other than this is anti science, anti truth, anti biological functionalism, anti white birth rates, anti nature, etc.”

Figure 4 is explicit in its misogyny and desire to subjugate women, comparing the importance of controlling white women to the need for water: “Is it good for the white tribe to drink water? If yes then we do it. Is it good for the white tribe to control their women? If yes then we do it.” The authors also court the taboo against Islam by utilizing it to solve the great replacement problem.

### Science talk

We found policy memes in threads discussing the appropriate times to have sexual relationships with girls. These debates were contextualized within the continued imaginings of a white ethno-state. For example, in a thread discussing age of consent, one user wrote, “what would happen if age of consent changed to 14?” to which another responded, “the white population would explode.” Another user added that the age of consent had to be coupled with marriage of white girls at young ages to prevent their inevitable desire to “race mix.” These threads exemplify theoretical imaginings of the white ethno-state that affirm the fetishization of virgins—and sex with children. They also highlight the preoccupation with countering replacement and protecting white women and girls from race mixing impulses.

In another thread, in response to an age of consent question, a user posted Figure 4 adding “whenever she can give a healthy birth to a healthy baby, there will never be other acceptable answers to this question.” Another commented, “12-14 on the average the increased danger of the mother dieing is mainly before and also just about on the level of 29 years of age.” Others contributed ideas about the need for proper medical care in the white ethno-state to ensure young women and girls produce healthy babies. Thus, even though Figure 4 is scientifically vague, commentators filled in the blanks with the specifics that they believed would counter replacement, circumvent white women’s innate untrustworthiness, and help create and maintain the white ethno-state. Pedophilia here—another taboo—is seen as a functional and logical necessity to maintain priorities about reproductive purity and limiting the contagion of feminism—both culturally and through retrovirus transmission.

**Figure 5.**
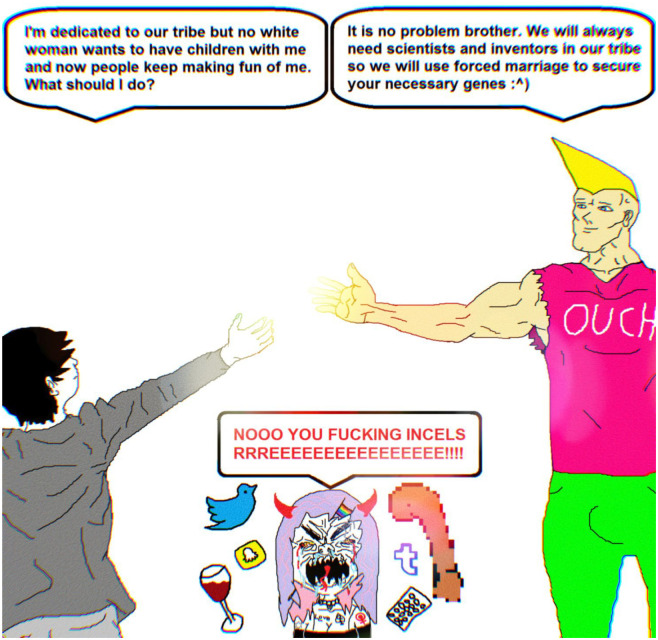
White Nationalist Chad and Incel Unite Meme.

Figure 4 also functioned to explicitly bridge Incels (self-proclaimed involuntary celibates) and “chads,” or men who have luck with women. For instance, one thread entitled “incels” began with a meme about ethno-states. The thread, in normal 4chan fashion, oscillated between true discussion, name calling, and meme posting. Throughout the usual noise, the conversation pulled toward the need for white ethno-states. In the middle of the thread, someone posted the following (Figure 5).

Figure 5 depicts chad 4chan users embracing their Incel brothers, against the screaming banshee that is presumably a woman. The thread continued in typical 4chan manner until someone posted the policy meme, commenting “this.”

Policy memes, like Figure 4, created the opportunity for elaborate theorizations of gender, which grew to include the fetishization of virgins and sexual desire for children, all within the meme’s science fan fiction universe. Explicitly, users connected Figure 4’s ideas to argue for sexual access to white girls to protect bloodlines and prevent white genocide. Thus, fantastical science, in combination with hyper-patriarchal policy ideas, serves as a jumping off point to solve demographic fears around population changes. As this is a public forum, all memes functioned as sounding boards for further discussion between idealists and pragmatists, allowing a wide range of opinions and resentments. Furthermore, policy memes, like Figure 4, worked as a bridge for different 4chan communities to reach out to the Incel community through the shared interest in virgins and subjugating women, both physically and sexually.

## 6. Conclusion

This article analyzed how 4chan users “scientifically” articulate gender resentment on the 4chan /pol/board. We have demonstrated the following two key findings: (1) the existence of a gendered, hate-based scientific discourse, “science fan fiction,” and (2) how gender science fan fiction is an outcome of the far-right cosmology. We further illustrated that they use this distinct scientific discourse to substantiate claims of white male supremacy, the fundamental untrustworthiness of women, and what this distrust means to their larger goals.

This article contributes to the growing body of research on the far-right and their use of the Internet by showing another tactic in the targeting of young men with the intent to radicalize (Marwick and Lewis, 2017). Specifically, our findings add nuance to understanding *how* 4chan users utilize anti-women, anti-feminism, pro-patriarchy “science” as a key discourse for shared grievances that may bring other Internet cultures, like Incels, into the far-right belief system. The article’s suggestion about overlaps between the far-right, white nationalists, and Incels provides intriguing details about how these movements use these online spaces and baroque theorizing to bridge movements through the guise of further redpilling. This should be further explored in future research.

This article also illustrates that there are important connective threads within the online far-right community: its shared hatred for white women, racist anxieties about demographic changes, and science (Ging, 2019). Fears over “the great replacement” ultimately reflect the inability to control human reproduction. Control over reproduction and the desired societal utopia this could produce represent the core policies in which gender plays both a biological and a social role, whether imagined by 4chan users or by the state.

The importance of this cannot be overstated, as panic over replacement, often stoked in these communities, has led to acts of violence. For example, the shooters in El Paso, Christchurch, Buffalo, and Poway, all posted manifestos on far-right message boards and cited the great replacement as a main motivator for their violence. The Christchurch shooter began his manifesto with, “It’s the birth rates.” Thus, to understand the far-right online cosmology is to reconcile with how it uses gendered discourse in discussions of reproducing the nation, male supremacy, and misogyny. In today’s modern age, this includes discourse in radical online spaces and Internet memes boasting outlandish ideas.

We are aware that the culture of 4chan is based in irony, sarcasm, and extreme onlineness (Kasimov, 2021). Although 4chan users can always claim they were joking or that the space is for the expression of all ideas, outrageousness and provocation are still values. Moreover, if these denials were true then the themes found in the secondary discourses would not have overlapped. Our findings suggest that there may be an effort to cover the entire /pol/board with misogynistic and virulently anti-women ideas, perhaps to recruit. Regardless of intent, this article shows that thread discussions become progressively more radicalized after the deployment of the science fan fiction memes.

This article contributes to larger scholarly conversations about how far-right forces engage science. The far-right’s treatment of science when discussing race involves the selective amplification of academic literature, drawing quasi credible connections between somewhat well-established scientific concepts and generating new knowledge within the framework of legitimate science (Panofsky et al., 2021). Our article demonstrates a different approach to gender that relies less on replicating the nuances and finesse of the related science, but instead on developing extravagant, vaguely scientific inspired accounts of gender differences, which are inherently related to racist ideologies.

The baroqueness of gender science fan fiction thus stands in deep contrast to the “sobriety” of basic racial theorization produced by the far-right. However, the two discourses share the connective thread that exemplifies the movement’s core values: white supremacy, the need for racial separation, and strictly enforced patriarchy. These two approaches signify two sides of far-right scientific discourse, both which rearticulate a particular and virulent racialized gender politics. Racialized politics of white nationalist movements and patriarchy, of course, is not new (Blee, 2002). However, the use of radical websites has given these movements an unprecedented platform for larger audiences and the ability to evolve their messaging. Future research should take a systematized and comparative approach to analyze the differences between far-right race science and discussions of gender difference and resentment.

Our finding about science fan fiction is very intriguing, as it demonstrates a reliance on science to be a legitimizing anchor to far-right arguments about the social world, no matter how improbable. Furthermore, while these 4chan users are “fans” of science, their pursuit of it resembles the technique of creative exploration that we find in fandom communities, rather than the technique of investigative inquiry found in science journalism, and so on. It also adds to the growing literature on toxic fandoms and conceptualizing white male supremacy as a fandom and how these communities function in far-right online spaces (Hodson and Gosse, 2022; Stanfill, 2020). Furthermore, we join this growing field’s call to take reactionary fandoms and science fan fiction seriously as it may function more like white nationalist fiction of the past, such as the *Turner Diaries* (often seen as the influence for the Oklahoma City bombing), in both its meaning and importance. Science fan fiction similarly touches on white nationalist cosmology, which has long mixed beliefs about racial purity, racial separation, demographic changes, strictly enforced patriarchy, and domination of women. Fiction writings are thus important discourses, as they may be key mobilizing agents for various far-right groups, especially those with a penchant for violence.

## Notes

1. We have censored racial slurs to not replicate and reproduce the original authors’ language.
